# Identification and genetic characterization of a novel *Orthobunyavirus* species by a straightforward high-throughput sequencing-based approach

**DOI:** 10.1038/s41598-019-40036-4

**Published:** 2019-03-04

**Authors:** Ohad Shifman, Inbar Cohen-Gihon, Adi Beth-Din, Anat Zvi, Orly Laskar, Nir Paran, Eyal Epstein, Dana Stein, Marina Dorozko, Dana Wolf, Shmuel Yitzhaki, Shmuel C. Shapira, Sharon Melamed, Ofir Israeli

**Affiliations:** 1 Department of Biochemistry and Molecular Genetics, Israel Institute for Biological Research, Ness Ziona, Israel; 2 Department of Infectious Diseases, Israel Institute for Biological Research, Ness Ziona, Israel; 3 Department of Biotechnology, Israel Institute for Biological Research, Ness Ziona, Israel; 4 Clinical Virology Unit, Hadassah Hebrew University Medical Center, Jerusalem, Israel; 5 Israel Institute for Biological Research, Ness Ziona, Israel

## Abstract

Identification and characterization of novel unknown viruses is of great importance. The introduction of high-throughput sequencing (HTS)-based methods has paved the way for genomics-based detection of pathogens without any prior assumptions about the characteristics of the organisms. However, the use of HTS for the characterization of viral pathogens from clinical samples remains limited. Here, we report the identification of a novel *Orthobunyavirus* species isolated from horse plasma. The identification was based on a straightforward HTS approach. Following enrichment in cell culture, RNA was extracted from the growth medium and rapid library preparation, HTS and primary bioinformatic analyses were performed in less than 12 hours. Taxonomical profiling of the sequencing reads did not reveal sequence similarities to any known virus. Subsequent application of *de novo* assembly tools to the sequencing reads produced contigs, of which three showed some similarity to the L, M, and S segments of viruses belonging to the *Orthobunyavirus* genus. Further refinement of these contigs resulted in high-quality, full-length genomic sequences of the three genomic segments (L, M and S) of a novel *Orthobunyavirus*. Characterization of the genomic sequence, including the prediction of open reading frames and the inspection of consensus genomic termini and phylogenetic analysis, further confirmed that the novel virus is indeed a new species, which we named Ness Ziona virus.

## Introduction

Viruses are a global source of significant health and economic burdens. Interactions between humans and animals are the paramount driving force for the ever-growing distribution and emergence of novel viruses. Other reasons for the spread of new viruses include climate change, globalization, social mobilization and intensive farming^1–3^. A novel virus might emerge in the form of an outbreak, and in this scenario, rapid diagnosis is of extreme importance for the selection of prevention and treatment strategies. However, such a diagnosis is not trivial, especially for RNA viruses, since the viruses in this category have small but highly variable genomes^4^.

Viruses can be identified by a wide range of techniques. Traditional methods rely on morphological characteristics observed by light microscopy or transmission electron microscopy (TEM) in various specimens such as cell cultures and fertilized eggs. Serology, as well as antibody-based diagnostics, allow to identify the virus and in some cases even at the species level. However, these methods provide only morphological clues, depend on the availability of an antiserum and, for the most part, are not strain specific^5^. In recent decades, molecular methods such as PCR, RT-PCR and microarrays, which are in most cases more sensitive than traditional techniques, have been used to complement and even replace traditional techniques. However, most of the molecular assays mentioned above are designed to be pathogen specific or are aimed at a limited group of infectious agents. The narrow scope of these methods significantly limits their ability to discover new, unknown pathogens and hampers our ability to reveal the full diversity of a given clinical specimen^6,7^.

High-throughput sequencing (HTS) technologies, developed in recent years, have substantially improved the capability of comprehensive detection of pathogens without any prior assumptions about the characteristics of the organisms (i.e., “unknown” samples). These massive parallel sequencing platforms can sequence mixtures of genetic materials from heterogeneous samples with high sensitivity and speed and at a lower cost per base than traditional Sanger sequencing^8,9^. In addition, HTS technologies, have benefits other than the improved detection of known and unknown pathogens in different samples. Among these benefits is the ability to detect nonculturable organisms as well as coinfection, drug resistance or response to therapy^10,11^.

The use of HTS for characterization of unknown viral pathogens in relevant clinical samples remains limited, primarily because of the large ratio between the genome sizes of hosts and pathogens. This limitation is especially true of blood-related samples, where white blood cells are abundant. Nonetheless, virus detection in clinical samples by HTS is starting to be increasingly used. Studies that have tried to identify unknown RNA viruses by using HTS have been conducted mostly in less-relevant clinical samples (i.e., brain) and provided diagnoses in shorter time frames because they did not require culture first, while other studies have typically obtained between tens to a few thousands of viral reads, a number that might be sufficient for resequencing against a model organism but not for characterizing novel viruses^12–20^.

In this study, an HTS-based approach was applied to identify the origin of a viral pathogen found to be present in plasma obtained from a hyperimmune horse^21^. To this end, we have established a straightforward procedure that includes virus enrichment in cell culture followed by RNA extraction from the growth medium, rapid library preparation, sequencing and in-depth data analyses. By following this procedure, we successfully identified and characterized a novel species belonging to the *Orthobunyavirus* genus.

## Results

Viral safety of biological products derived from equine plasma is verified by testing the starting material of the process (hyperimmune horse plasma) for viral contamination^22^ and by assessing the efficacy of the entire production process to inactivate infectious viruses^23^. As part of such a process, batches of plasma obtained from a hyperimmune horse, producing anti-botulinum antibodies, were routinely inspected for viruses by incubating the plasma in Vero and BHK cell lines and monitoring them for cytopathic effect (CPE). In one batch (H234), the presence of a virus was detected. The hyperimmune horse was closely monitored and did not show any clinical manifestation indicating viral infection. Although this batch of plasma was rejected and was not further considered for production purposes, identification and characterization of the virus present in the sample is of major importance for process assessment.

For this purpose, we applied a straightforward procedure that has been established in our lab for the detection and identification of unknown viruses. This procedure involves preliminary virus enrichment in cell culture, which is followed by RNA extraction from the growth medium, rapid library preparation, HTS and bioinformatic analyses.

### Virus isolation and enrichment

**Figure 1:**
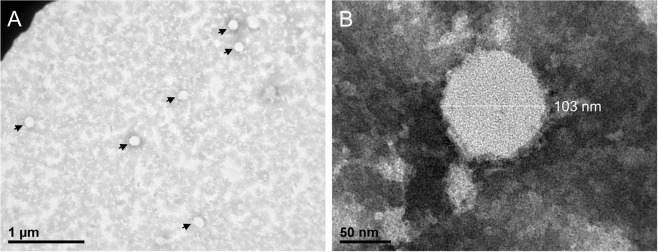
TEM images of viral particles from the supernatant of Vero cells infected with horse plasma. The supernatant was inactivated by Karnovsky solution and negatively stained with 1% phosphotungstic acid. (**A**) Low magnification (bar represents 1 µm). Viral particles are indicated by arrows. (**B**) High magnification (bar represents 50 nm). Representative viral particle with a diameter of ~100 nm.

Vero cells were infected with the virus-containing plasma and incubated for 6 days until a massive CPE was observed, which was followed by an additional round of viral enrichment. The viral titer in the supernatant was determined by a plaque assay to be 5.7 × 10^7^ pfu/ml. TEM revealed the presence of spherical particles that were approximately 100 nm in diameter and had jagged edges (Fig. 1). These results confirmed the presence of high-titer viruses in the sample. Nevertheless, the morphology observed was not pathognomonic for virus identification and could be associated with several viral families.

### RNA extraction, library preparation and sequencing

The SMARTer Pico RNA Kit we used in this study enables rapid and direct library preparation from very low amounts of starting material. This procedure, with adjustments made in our lab (see Methods), was performed by a single-tube process that involved RNA fragmentation, random priming, first- and second-strand synthesis, and depletion of the ribosomal cDNA originating from the Vero cells. The libraries were sequenced as single reads of 60 nucleotides by a MiSeq instrument, and the sequencing resulted in over 10 million passed-filter reads. The whole process, including RNA extraction, library preparation and sequencing, lasted less than 12 hours. RNA from noninfected Vero cells served as a negative control for the above procedure.

### Primary analysis of sequencing results

To identify the virus in the sample, we initially utilized two rapid bioinformatic tools, MetaPhlAn2 and Pathoscope, which profile obtained reads by comparing the reads to databases of microbial genomic sequences. MetaPhlAn2 maps reads against a database of predefined clade-specific genetic markers originating from bacterial, fungal and viral genomes, while Pathoscope uses various databases of whole-genome sequences, containing over 10,000 complete bacterial, fungal and viral genome sequences. However, no significant viral hits were found by either computational method, indicating that the viral sequence is not present in the databases currently used for analysis. Four percent of the reads could be attributed to bacterial and fungal sequences or to controls that were added to the sample (PhiX174 and carrier RNA). Approximately 78% of the reads were mapped to the green monkey (*C. sabaeus*) genome, while in the negative control sample, over 90% of the reads were mapped, implying that these reads originated from the Vero cells. Notably, a significant portion of the reads (18%) could not be assigned to sequences in the databases. We hypothesized that the as-yet-unidentified viral sequences might be among these reads. To determine the origins of these unmapped reads, we applied a *de novo* assembly approach in order to obtain long continuous sequences (contigs) and reconstruct the viral sequence. Such contigs may then be subjected to sequence similarity searches against the entire NCBI nr/nt nucleotide collection and to characterization of the genome sequence of the virus.

### *De novo* assembly

**Figure 2:**
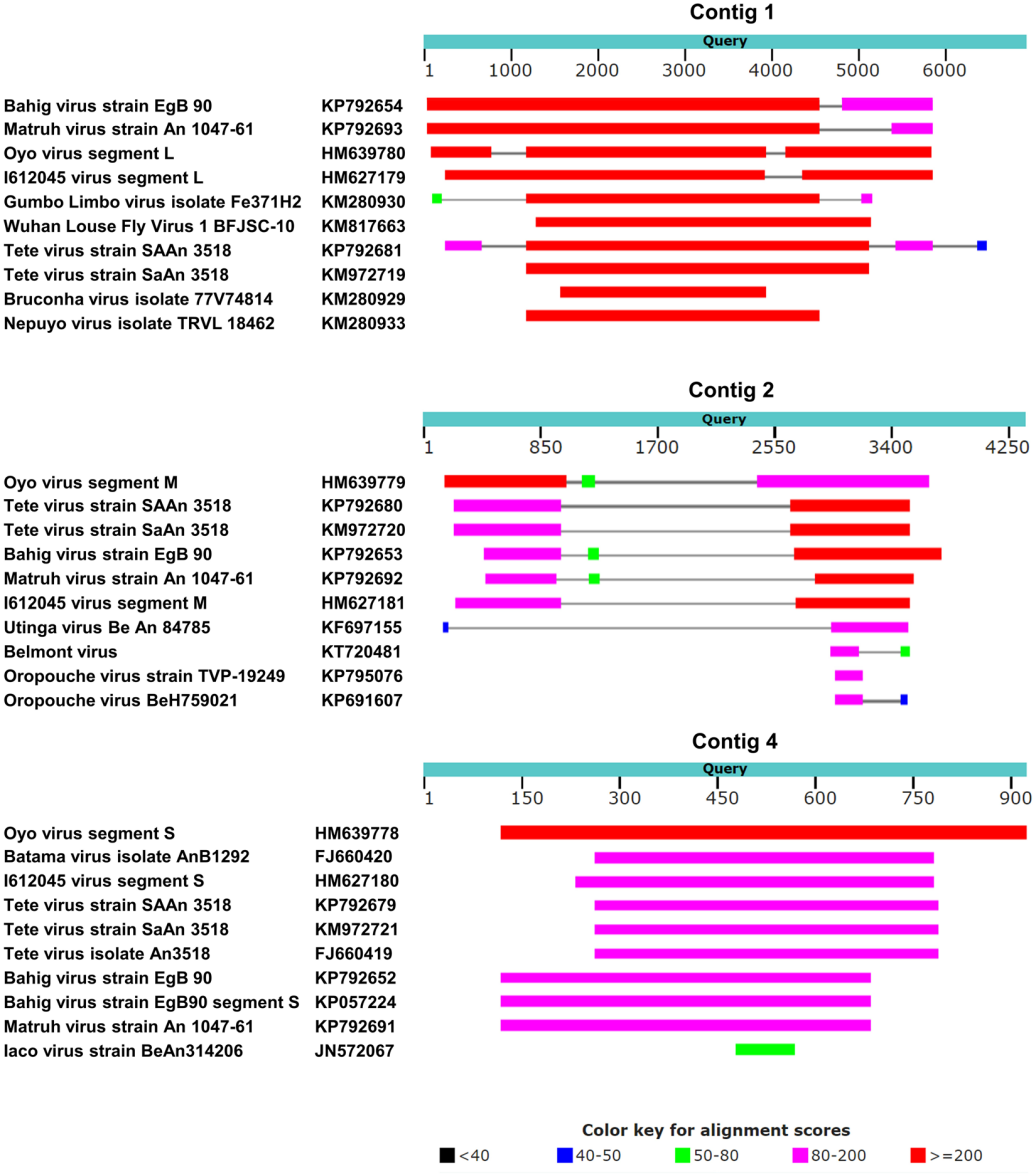
Top BLAST hits of the Velvet-assembled contigs. BLAST analyses were performed using the Velvet-assembled contigs as queries and the nr/nt nucleotide collection as a database. The top ten hits that were identified for contigs 1, 3 and 4 by BLAST are presented as a graphical overview, where for each hit, the locations and lengths of the homologies that were found are shown using a ruler (in bases) based on the query length. The homologies are color coded according to the BLAST scores.

Following the removal of sequences originating from the Vero host cells, the remaining reads were subjected to analysis by the Velvet assembler using optimized parameters obtained automatically from the multithreaded script VelvetOptimiser (see Methods). The assembly resulted in eight contigs with lengths ranging from 255 to 6834 bases (Table 1). In the control sample no relevant contigs were found. Each contig was subjected to a sequence similarity search against the nr/nt nucleotide collection using BLAST. Of these eight contigs, four (contigs 5 to 8) were highly similar to rRNA sequences, while one contig (contig 2) matched the PhiX174 genome. BLAST results for contigs 1, 3 and 4 did not show a complete match with any sequence in the database, with the best hits exhibiting less than 70% identity; contigs 1, 3 and 4 most resembled the L, M and S genomic segments, respectively, of viruses from the *Orthobunyavirus* genus (Fig. 2). Notably, the lengths of the contigs were in best agreement with the typical lengths of the *Orthobunyavirus* genomic segments and substantially differed from those of other genera in the *Bunyavirales* order (Table 2). These results are consistent with the assignment of the generated sequences to the *Orthobunyavirus* genus, while the low sequence homology to known *Orthobunyavirus* sequences suggests the classification of this virus as a new species. Notably, the morphology of the virus, as observed by TEM, is consistent with this classification. We named the virus Ness Ziona virus (NZV) after the location it was discovered in. To verify the presence of NZV in the horse plasma, we designed a specific NZV RT-PCR test based on the assembled NZV S genomic segment. The test was conducted on the original plasma before culturing (H234) and after culturing (H234-Vero). A plasma sample taken from the same horse before infection (H210) was used as a control. The presence of the expected 162-bp band confirmed the existence of NZV in the infected H234 plasma and its absence from the preinfection sample (Supplementary Fig. 3).

**Table 1:** Sizes and identification of the Velvet-assembled contigs.

Contig no.	Contig length (bases)	BLAST identification
1	6834	?
2	5430	PhiX174
3	4351	?
4	913	?
5	555	rRNA
6	547	rRNA
7	357	rRNA
8	255	rRNA

**Table 2:** Genomic segment (L, M and S) lengths (in kilobases) of the type species of the *Orthobunyavirus* genus and related genera of the *Bunyavirales* order.

Family	Genus	Type species	L	M	S
*Peribunyaviridae*	*Orthobunyavirus*	*Bunyamwera orthobunyavirus*	6.9	4.5	1.0
*Peribunyaviridae*	*Herbevirus*	*Herbert herbevirus*	7.4	2.7	1.1
*Feraviridae*	*Orthoferavirus*	*Ferak orthoferavirus*	6.9	4.3	1.5
*Hantaviridae*	*Orthohantavirus*	*Hantaan orthohantavirus*	6.5	3.6	1.7
*Jonviridae*	*Orthojonvirus*	*Jonchet orthojonvirus*	6.9	5.4	1.7
*Nairoviridae*	*Orthonairovirus*	*Dugbe orthonairovirus*	12.3	4.9	1.7
*Phasmaviridae*	*Orthophasmavirus*	*Kigluaik phantom orthophasmavirus*	6.7	2.8	2.2
*Phenuiviridae*	*Goukovirus*	*Gouleako goukovirus*	6.4	3.2	1.1
*Phenuiviridae*	*Phasivirus*	*Badu phasivirus*	6.9	3.9	1.5
*Phenuiviridae*	*Phlebovirus*	*Rift Valley fever phlebovirus*	6.4	3.9	1.7
*Tospoviridae*	*Orthotospovirus*	*Tomato spotted wilt orthotospovirus*	8.9	4.8	2.9

Taxonomical data are based on the Master Species List (MSL31) of the International Committee on Taxonomy of Viruses (ICTV)^28^. Type species and their genome sizes were taken from the MSL31 and the NCBI genome website (https://www.ncbi.nlm.nih.gov/genome/).

### Refinement of the genome assembly

**Figure 3:**
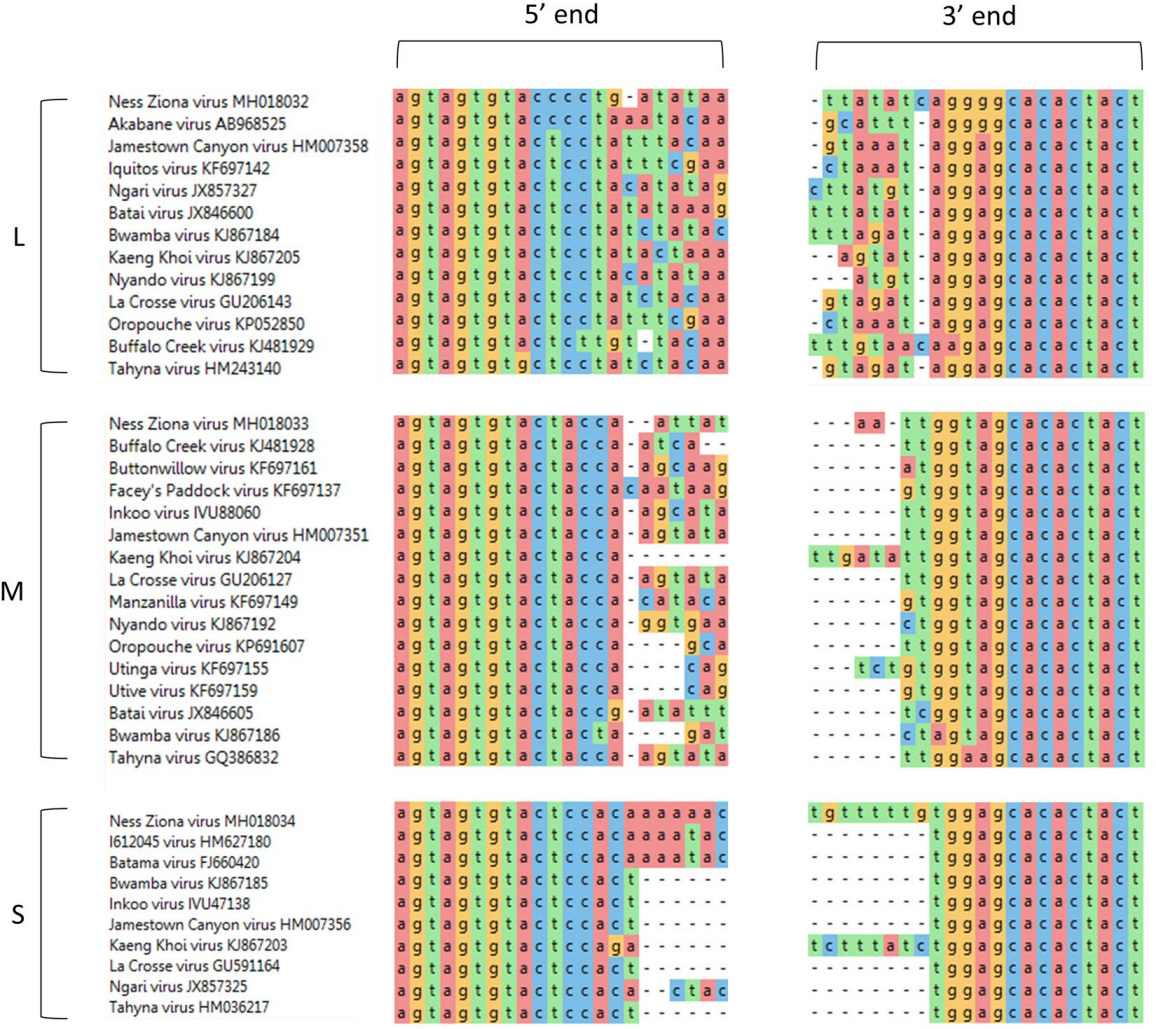
Comparison of the 5′ and 3′ ends of *Orthobunyavirus* genomic segments. The genomic sequences (L, M or S) of the indicated viruses, which belong to the *Orthobunyavirus* genus, were aligned by MegAlign Pro software using the MUSCLE algorithm and default parameters. The first 22 bp (5' end) and last 22 bp (3' end) of the alignment are presented using a color-coded background. Accession numbers of each virus are indicated following the name of the virus.

*Orthobunyavirus* members are known to possess characteristic short consensus sequences at their termini (5′ and 3′)^24^. In-depth analysis of the NZV sequence revealed that some of the segment termini lacked this consensus sequence. We therefore applied an additional algorithm for *de novo* assembly using the SPAdes tool. This analysis resulted in contigs that were similar to but longer than those obtained from the Velvet algorithm, and these contigs included the conserved sequences at all termini, which were flanked by short sequences that were apparently host-derived^25–27^. To further refine and verify the sequences of the three segments, we realigned the original sequencing reads to the longer sequences obtained from SPAdes and removed low-quality alignments. This procedure resulted in high-quality, manually curated, complete genomic sequences of the three segments of NZV. The complete genomic sequences were annotated and deposited in the NCBI GenBank (accession numbers MH018032, MH018033, and MH018034 for the L, M, and S segments, respectively). Comparison of the 5′ and 3′ ends of each of these NZV segments revealed that the novel virus contains a similar consensus sequence as other *Orthobunyavirus* species (Fig. 3). These findings support the classification of NZV as an *Orthobunyavirus*.

**Figure 4:**
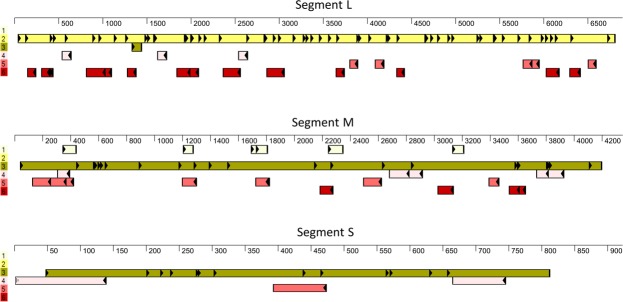
Predicted open reading frames of the Ness Ziona virus (NZV). Open reading frames (ORFs) were predicted using SeqBuilder Pro for the 3 genomic sequences (L, M and S) of the NZV. The potential ORFs (longer than 25 amino acids and containing a start codon) that were identified for each genomic segment are shown. The color code indicates the frame of each ORF, where 1 to 3 and 4 to 6 are the forward and reverse frames, respectively. The ruler (in bases) indicates the positions of the ORFs on the genomic segment. Filled triangles and vertical black lines indicate potential start and stop codons, respectively.

To further characterize the viral genomic sequence, we predicted potential open reading frames (ORFs) in the sequence. Each segment was found to encode a long ORF spanning nearly the entire segment (Fig. 4), as expected for an *Orthobunyavirus* member^24^.

### Phylogenetic analyses

**Figure 5:**
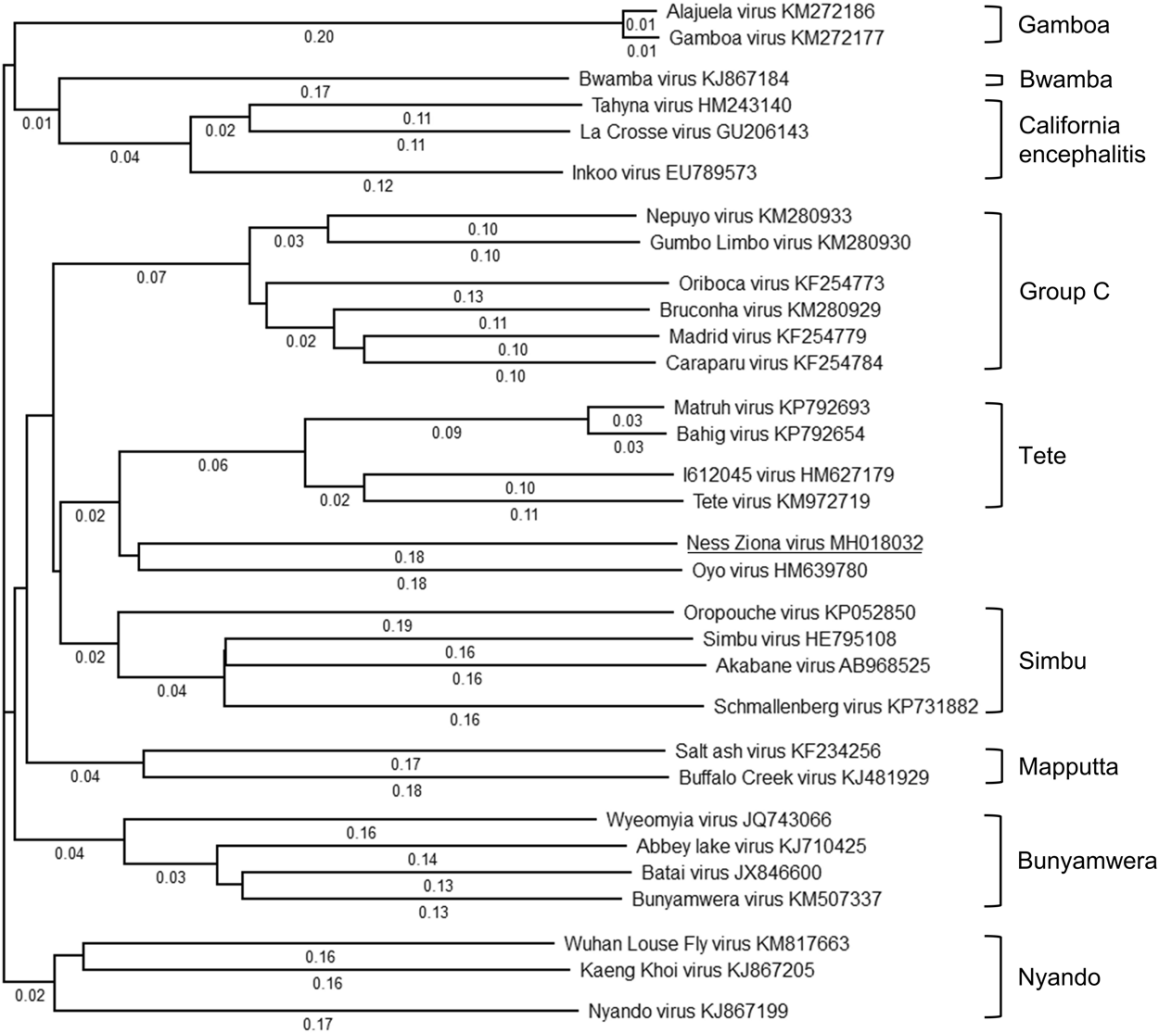
L-segment-based phylogenetic tree of the Ness Ziona virus (NZV) and other *Orthobunyavirus* members. L-segment sequences of NZV and the indicated viruses were aligned by MegAlign software using the MUSCLE algorithm with default parameters. The resulting phylogenetic tree based on this alignment is presented. NZV is underlined. The calculated distance for each branch is indicated. The accession number of each virus is noted after the name of the virus. Serogroups of the viruses are noted to the right of the figure.

*Orthobunyavirus* is a genus comprising 48 recognized species and more than 170 named viruses^28,29^. Viruses of this genus are typically arthropod borne but are diverse in terms of geographical distribution, principal vectors and hosts. Currently, most of the viruses of this genus are classified into 18 serogroups^29^. To determine the evolutionary relationship of the newly identified NZV to the known species in the *Orthobunyavirus* genus, phylogenetic analyses were conducted on the full-length genomic segments of the closest species that were derived from the BLAST analyses and representative *Orthobunyavirus* species from different serogroups (Fig. 5, Supplementary Figs 1 and 2). These phylogenetic results were consistent with the serogroup classification. The phylogenetic analyses emphasized that NZV is a distinct species that is most similar to the Oyo virus, an unclassified *Orthobunyavirus*^30^, and is in the same branch as viruses of the Tete serogroup.

## Discussion

In this study, we established a rapid and straightforward protocol based on HTS for the identification of unknown RNA viruses from cell culture-enriched clinical samples. Once sufficient viral material is available from culture, our protocol is applicable. The main limitation in HTS-based identification, especially from relevant clinical specimens, such as whole blood, plasma or serum samples, is the high host nucleic acid content, which typically masks the relatively low viral nucleic acid content. Previous protocols^31–34^ that utilized HTS to characterize unknown RNA viruses are relatively laborious and time-consuming and are based mostly on tissues containing high viral titers that are obtained postmortem^13,15,16^. Cell culture enrichment can overcome this problem to a certain extent; however, even after viral enrichment, the host background can be dominant. Cell culture rRNA, which amounts for more than 90% of the total RNA in the culture, significantly contributes to the high background^35^.

In the protocol described in this study, the clinical sample was first grown in cell culture until a high viral titer was obtained, and the growth medium was then harvested. Previous experiments conducted by our group demonstrated that although the growth medium fraction contained less RNA than the cell pellet, the viral RNA was enriched in that fraction (data not shown). We used the QIAamp Viral Mini Kit for extraction and purification of the RNA since this is most suitable kit for the subnanogram amounts of RNA commonly present in the growth medium fraction. We found that carrier RNA, which was added to improve the yield of the extraction, normalized the amount of initial RNA needed for library preparation and did not interfere with downstream processes, improving upon previous protocols that included a carrier RNA depletion step^32–34^. We chose to prepare the HTS libraries using the SMARTer Pico RNA Kit for several reasons. The initial RNA amount requirements for this kit are among the lowest for commercial kits, and the RNA concentrations obtained from the RNA extraction step are within the required range. This kit uses random (and not oligo-dT) priming for first-strand synthesis and proprietary SMART technology for template switching and second-strand synthesis. These unique features favor the probability of obtaining full-length transcripts, even from nonpolyadenylated viruses, as in our case. In addition, depletion of the ribosomal cDNA originating from host cells is integrated into the kit and allows whole library preparation to be performed in a single tube and in a relatively short amount of time.

The libraries were sequenced as single-end reads of 60 nucleotides. Sequencing reads of 60 bases enable short run times (<5 hours) and, in our hands, are sufficient for identification and characterization of RNA viruses, which have short and simple genomes, even when *de novo* assembly and reconstruction of the genome is required. The whole process, including RNA extraction, library preparation, sequencing and primary bioinformatic analysis, can be completed in less than 12 hours.

To identify the virus present in the horse plasma, we first employed two programs that specialize in rapid and accurate taxonomic profiling of repertoires of microbial organisms in sequenced samples. Nevertheless, a prerequisite for successful identification of a microbial agent by these tools is the existence of the sequence of the organism in the databases used to analyze the data. In the current study, NZV was discovered to be a novel *Orthobunyavirus* and thus could not be recognized by these profiling tools. We therefore applied a complementary approach based on *de novo* assembly that did not require any prior knowledge of the sequences of the organisms present in the sample. We used two different algorithms for the *de novo* assembly, namely, Velvet and SPAdes, which produced similar results, although Velvet was somewhat more stringent and thus gave slightly shorter contigs. Three of the resulting contigs could be assigned to the L, M and S segments of NZV. After a refinement step, where the contigs were used as references to map the reads, and after filtering out the low-quality alignments, we obtained the complete sequences of the three segments of the virus. Determination of whole-genomic sequences is the gold standard in genomic characterization and allowed in-depth characterization of NZV. The characterization included the prediction of ORFs, identification of the consensus sequences of the genomic termini, and phylogenetic classification; the phylogenetic classification closely resembled the serogroup classification. Notably, segments L and S showed high homology with other *Orthobunyavirus* members throughout the segments, while segment M exhibited lower homology in the central region and higher homologies in the flanks (Fig. 2). Segment M of *Orthobunyavirus* members typically encodes for three polypeptides, Gn, NSm and Gc, which are cotranslationally cleaved. The central region of the M segment encompasses the predicted NSm sequence. The low homology found in the NZV predicted NSm sequence might indicate the uniqueness of the virus and further supports the concept that this is a new member of the *Orthobunyavirus* genus.

Overall, we obtained compelling evidence indicating that we have identified a novel species, which belongs to the *Orthobunyavirus* genus and is closely related to the Oyo virus and to members of the Tete serogroup. Further elucidation of NZV biology necessitates further experimental research, which is beyond the scope of this report.

From this study, we infer that NZV can asymptomatically infect horses. The virus culture is available for further characterization. However, given that members of the *Orthobunyavirus* genus have been reported to infect other mammals, including cattle, sheep, pigs and humans, we cannot exclude the possibility that this virus can infect additional hosts^24^. Moreover, we cannot determine which vectors are involved, since there are various documented vectors of *Orthobunyavirus*, including mosquitoes, flies and ticks^24^.

In recent years, several *Orthobunyavirus* species have been found to be endemic to Israel^36,37^. These viruses, including the Shuni and Akabane viruses, belong to the Simbu serogroup and do not resemble NZV, suggesting that there is no relation or reassortment between the viruses.

The identification and characterization of the novel NZV *Orthobunyavirus* described in this study demonstrate the power of HTS, combined with bioinformatic tools, to rapidly identify and gain in-depth understanding of unknown and novel viruses in clinical samples. In a scenario of novel viral emergence, straightforward diagnosis, such as that described in this study, could be applied and be of utmost importance and value.

## Methods

### Ethics statement

All animal experiments were performed in accordance with the Israeli law and were approved by the Ethics Committee for Animal Experiments at the Israel Institute for Biological Research.

### Virus isolation

Vero cells (ATCC CCL-81) were routinely maintained in Dulbecco’s modified Eagle’s medium (DMEM) supplemented with 10% fetal calf serum (FCS), 2 mM L-glutamine, 0.1 mg/ml streptomycin, 100 units/ml penicillin, 1.25 units/ml nystatin and MEM-Eagle nonessential amino acids (Biological Industries). Vero cells were plated in T-25 flasks to obtain a monolayer after 24 hours of incubation. For infection, the culture media was aspirated, and the cells were washed twice with DMEM to reduce the FCS level. Horse plasma was diluted 1:50 in DMEM and overlaid on the Vero monolayer at a minimal volume (1 ml per T-25 Flask) for 1 hour at 37 °C on a reciprocal rocker. After 1 hour, the medium was aspirated, and fresh medium containing DMEM, 2% FCS, MEM-Eagle nonessential amino acids, 2 mM L-glutamine, 0.1 mg/ml streptomycin, 100 units/ml penicillin, 1.25 units/ml nystatin and 10 mM HEPES buffer was added. Six days post infection, when a massive CPE was evident, the culture medium was collected, and cell debris was removed by centrifugation (1700 RCF, 5 min). The collected medium was used to infect fresh Vero monolayers as described above. Five days post infection, when a CPE was observed, the culture medium was collected and used for analyses.

Viral titers were determined as pfu/ml using a plaque assay on Vero monolayers as previously described^38^.

### Transmission electron microscopy imaging

For TEM analysis, the supernatant from Vero cells infected with the horse plasma sample was inactivated and fixed using Karnovsky solution (4% paraformaldehyde and 2.5% glutaraldehyde) for 30 min at 25 °C, which was followed by an additional 30-min incubation at 25 °C^39^. Ten microliters of the inactivated supernatant was applied on 300-mesh carbon-coated copper TEM grids treated previously with 1% alcian blue. The samples were incubated for 10 min, and excess liquid was blotted using Whatman paper. The grids were washed three times with distilled water and stained with 1% phosphotungstic acid. After air-drying, the samples were visualized using an FEI Tecnai T12 microscope operated at 120 kV and equipped with a Gatan ES500W Erlangshen camera. Scaling was performed using a standard of known size, which was measured at different magnifications.

### RNA extraction and purification

RNA was purified from 140 µl of the cell culture supernatant by a QIAamp Viral RNA Mini Kit (Qiagen) according to the manufacturer’s protocol, using 60 µl of AVE buffer for elution. As a control, RNA was extracted as mentioned above from noninfected cells. RNA was quantified using a Qubit fluorometer with the Qubit RNA HS Assay Kit (Invitrogen). The concentration of the purified RNA was 6 ng/µl (and 5 ng/µl for the control), which is sufficient for library preparation using the SMARTer Pico RNA Kit. BioAnalyzer (Agilent) analyses utilizing the RNA HS kit did not provide a relevant RNA integrity number (RIN) because the major RNA peak was generated from the carrier RNA, which is added as part of the QIAamp Viral RNA Mini purification procedure.

### Library preparation and high-throughput sequencing

A SMARTer Stranded Total RNA-Seq Kit - Pico Input Mammalian (TaKaRa Bio) was used for library construction prior to sequencing on a MiSeq instrument (Illumina). This kit combines three technologies—SMART (switching mechanism at the 5′ end of RNA template) technology, LNA (locked nucleic acid), and a ribosomal cDNA depletion method—and the procedure can be completed in 5 hours.

Five nanograms of the extracted RNA was used for fragmentation at 94 °C for 4 min. First-strand synthesis was performed using SMARTer oligonucleotides, a template-switching oligo mix, partial Illumina adaptor sequences, and SMARTScribe reverse transcriptase at 42 °C for 90 min and then at 70 °C for 10 min. The cDNA was then amplified by 5 cycles of PCR with SeqAmp DNA polymerase and adapters for Illumina sequencing (with specific barcodes) and purified by two rounds of clean-up with 1 × AMPure XP beads (Agencourt). Ribosomal cDNA was depleted by a ZapR-mediated process, in which the library fragments originating from rRNA (18S and 28S) and mitochondrial rRNA (m12S and m16S) are cleaved by ZapR in the presence of R-Probes (which are mammalian-specific). These R-Probes were hybridized to ribosomal RNA and mitochondrial rRNA sequences for 60 min at 37 °C and then for 10 min at 72 °C. The depleted cDNA fragments were further amplified with universal Illumina primers for 16 PCR cycles. Lastly, the PCR products were purified once again by a single round of clean-up with 1 × AMPure XP beads to yield the final cDNA library. The library was sequenced in single-read mode (60 nucleotides) on a MiSeq instrument using the V2 Kit.

### Bioinformatic analyses

Sequencing reads were trimmed by 3 bases at the 5′ ends, and reads originating from the Vero cells were filtered out using SAMtools^40^ against the *Chlorocebus sabaeus* genome. The reads were subjected to taxonomical profiling with MetaPhlAn2^41^ using the default settings. Subsequent taxonomical profiling was performed with Pathoscope 2.0^42^ using an in-house-constructed target genome database containing all complete bacterial, viral and fungal genomes from the National Center for Biotechnology Information (NCBI) database (5414, 5777 and 3451 genomes, respectively). The reads were aligned to these genome sequences using the Bowtie2 algorithm^43^ with default Pathoscope parameters. *De novo* assembly of the reads was performed by SPAdes, with default parameters^44^, and Velvet^45^, using VelvetOptimiser^46^ to determine the optimized parameters for the assembly.

Multiple alignments and phylogenetic analyses were conducted by MegAlign Pro software using the MUSCLE algorithm with default parameters. ORFs were predicted with SeqBuilder Pro software using the following parameters: minimal length of 25 amino acids and beginning with a start codon. MegAlign Pro and SeqBuilder Pro are part of the Lasergene Genomics Suite, version 15 (DNASTAR).

BLAST analyses were conducted (on 04/Feb/2018) with the NCBI web interface (https://blast.ncbi.nlm.nih.gov/Blast.cgi), using the BLASTN algorithm optimized for “somewhat similar sequences (BLASTN)” and using the nucleotide collection (nr/nt) as a database.

### NZV detection by RT-PCR

Primers for RT-PCR (Forward: TCTGCTGGTGATGATGGATTAAA, Reverse: CATCTCACTTTTGTTTCTTCCTCTCA) were designed using Primer Express 3.0 (Applied Biosystems) based on the NZV S segment. RT-PCR assays were conducted in 25-µl reactions using a SensiFAST™ Probe Lo-ROX One-Step Kit (Cat# BIO-78005, BIOLINE) as follows: 5 µl of the purified RNA were added to 20 µl of reaction mix composed of 12.5 µl of 2 × SensiFAST™ Probe One-Step mix, 1.25 µl of molecular-grade H_2_O, 2.75 µl of the forward and reverse primers (at final concentration of 0.55 µM), 0.5 µl of RiboSafe RNase Inhibitor (provided with the kit) and 0.25 µl of reverse transcriptase. The RT-PCR thermal cycle was as follows: 48 °C for 20 min, 95 °C for 2 min and 45 cycles of 94 °C for 15 sec and 60 °C for 35 sec. Fifteen microliters of each RT-PCR reaction was mixed with 3 µl of 6 × Gel Loading Dye (Cat# B7025S, New England BioLabs) and subjected to 2%-agarose/ethidium bromide gel electrophoresis. The agarose gel was then visualized under UV light using a Bio-Rad Gel Doc 2000 gel documentation system. A 50-bp DNA ladder (Cat# N3236L, New England Biolabs) was used for RT-PCR product size determination.

## Supplementary information

Supplementary Information

## Data Availability

The authors declare that the raw data will be available on demand.
