# Supplementary material for: Identification and genetic characterization of a novel Orthobunyavirus species by a straightforward high-throughput sequencing-based approach
Source: Sci Rep. 2019 Mar 4;9:3398. doi: 10.1038/s41598-019-40036-4 (PMC6399452; doi:10.1038/s41598-019-40036-4)
Supplement: Supplementary file 1 — Supplementary Information [file 41598_2019_40036_MOESM1_ESM.pdf]

Identification and genetic characterization of a novel *Orthobunyavirus* species by a straightforward high-throughput sequencing-based approach

Ohad Shifman[a,1], Inbar Cohen-Gihon[a,1], Adi Beth-Din[a], Anat Zvi[a], Orly Levy[b], Nir Paran[b], Eyal Epstein[c], Dana Stein[a], Marina Dorozko[d], Dana Wolf[d], Shmuel Yitzhaki[e], Shmuel C. Shapira[e], Sharon Melamed[b], Ofir Israeli[a,*]

[a] *Department of Biochemistry and Molecular Genetics, Israel Institute for Biological Research, Ness Ziona, Israel*

[b] *Department of Infectious Diseases, Israel Institute for Biological Research, Ness Ziona, Israel*

[c] *Department of Biotechnology, Israel Institute for Biological Research, Ness Ziona, Israel*

[d] *Clinical Virology Unit, Hadassah Hebrew University Medical Center, Jerusalem, Israel*

[e] *Israel Institute for Biological Research, Ness Ziona, Israel*

* *Corresponding author.*

E-mail address: *ofiri@iibr.gov.il* *(O. Israeli).*

[1] These two authors contributed equally to this paper.

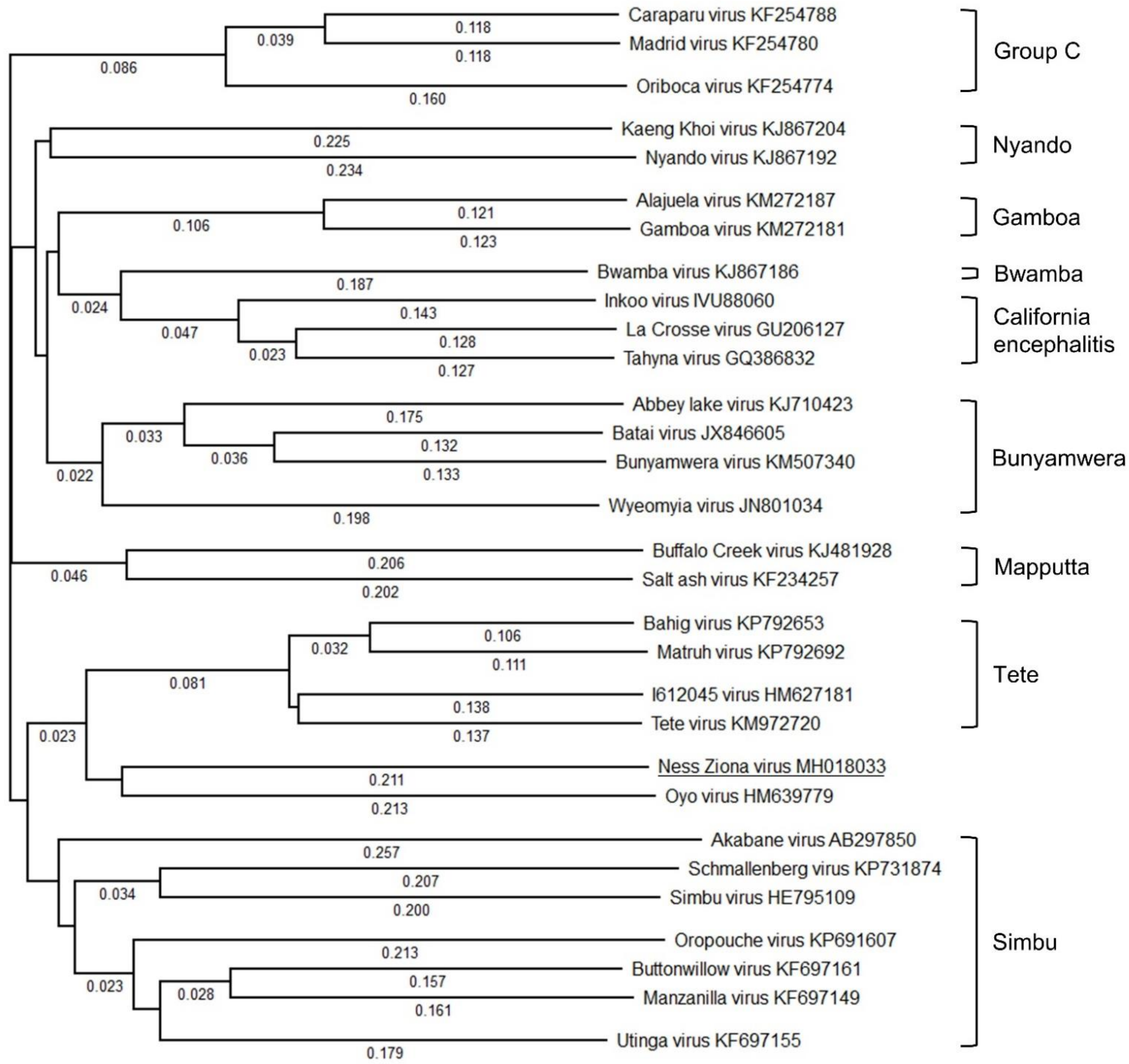

Supplementary Fig. 1. M-segment-based phylogenetic tree of the Ness Ziona virus (NZV) and other *Orthobunyavirus* members.

M-segment sequences of NZV and the indicated viruses were aligned by MegAlign software using the MUSCLE algorithm with default parameters. The resulting phylogenetic tree based on this alignment is presented. NZV is underlined. The calculated distance for each branch is indicated. The accession number of each virus is noted after the name of the virus. Serogroups of the viruses are noted to the right of the figure.

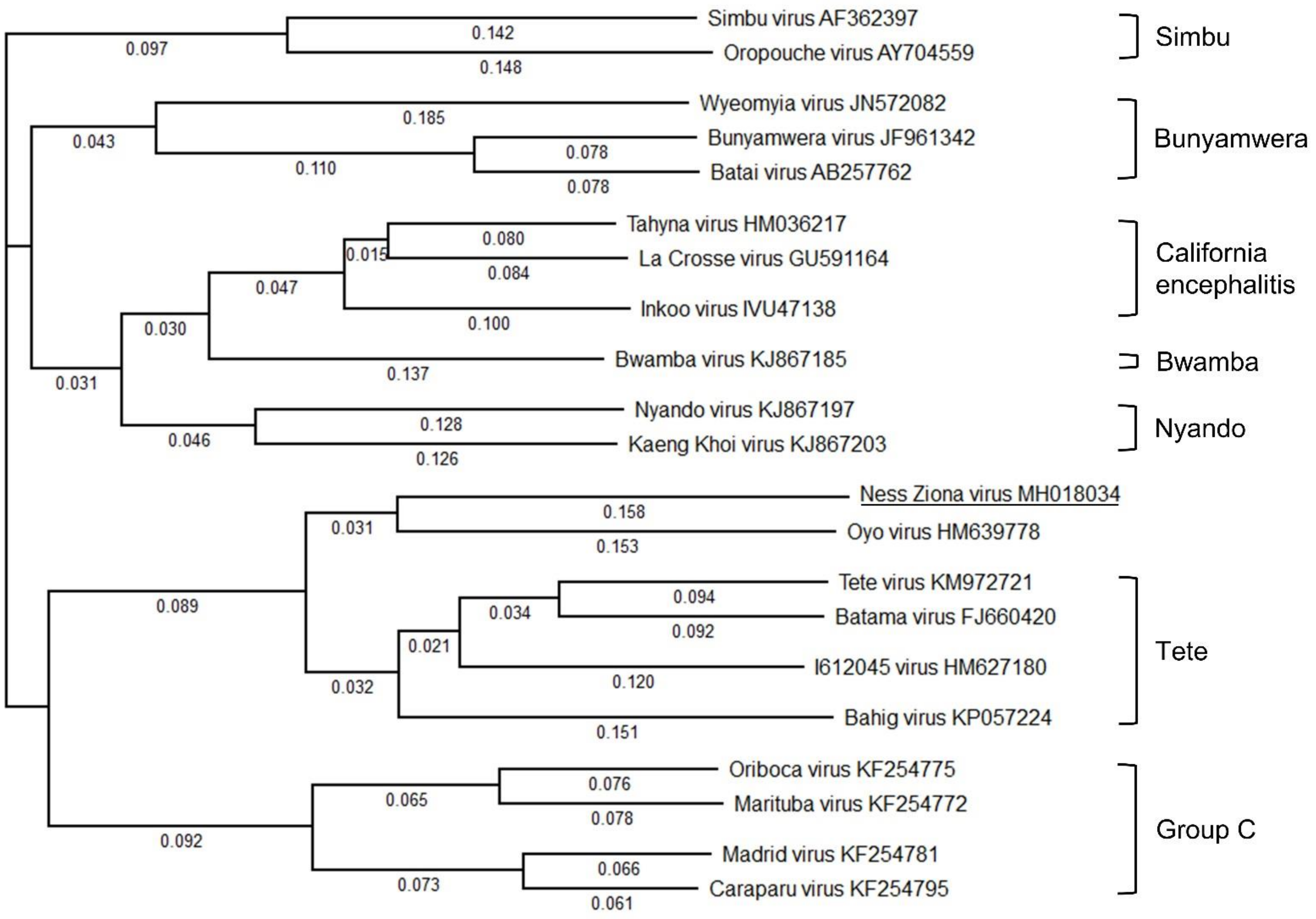

Supplementary Fig. 2. S-segment-based phylogenetic tree of the Ness Ziona virus (NZV) and other *Orthobunyavirus* members.

S-segment sequences of NZV and the indicated viruses were aligned by MegAlign software using the MUSCLE algorithm with default parameters. The resulting phylogenetic tree based on this alignment is presented. NZV is underlined. The calculated distance for each branch is indicated. The accession number of each virus is noted after the name of the virus. Serogroups of the viruses are noted to the right of the figure.

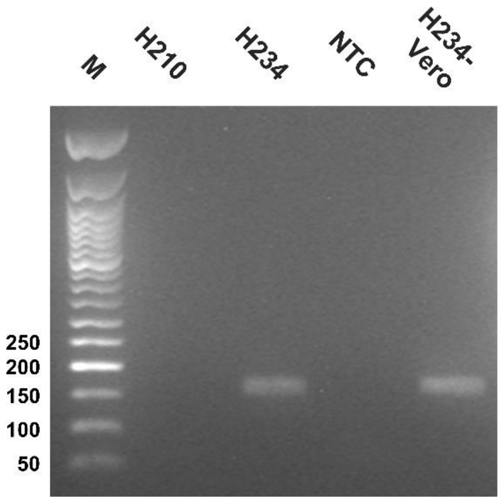

Supplementary Fig. 3. NZV detection in horse plasma by RT-PCR.

RNA was purified from two horse plasma batches, before (H210) and after (H234) infection, and from the postculture supernatant of H234 (H234-Vero). The RNA was subjected to RT-PCR using primers specific for NZV followed by gel electrophoresis. An RT-PCR reaction with no added sample (NTC) served as a negative control. A 50-bp DNA ladder size marker (M) was used to determine the PCR product lengths. Sizes (in bp) of the marker bands are indicated to the left of the gel.

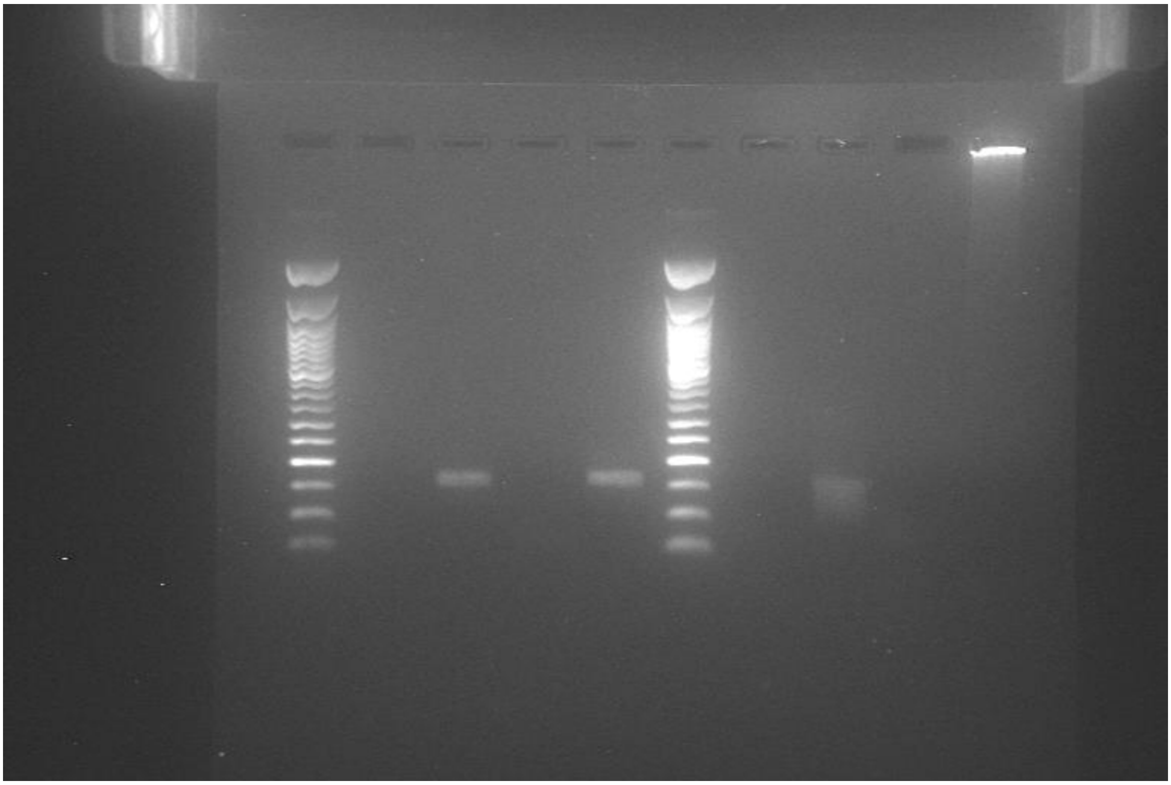

Supplementary Fig. 4. The full-length agarose gel used for Supplementary Fig. 3.
